# Examining Motivations to Play Pokémon GO and Their Influence on Perceived Outcomes and Physical Activity

**DOI:** 10.2196/games.8048

**Published:** 2017-10-24

**Authors:** Oriol Marquet, Claudia Alberico, Deepti Adlakha, J Aaron Hipp

**Affiliations:** ^1^ Department of Parks, Recreation and Tourism Management Center for Geospatial Analytics North Carolina State University Raleigh, NC United States; ^2^ School of Natural and Built Environment Queen’s University Belfast Belfast United Kingdom

**Keywords:** Pokémon GO, physical activity, excercise, exergames, gaming outcomes, games, recreational, motivation

## Abstract

**Background:**

Pokémon GO is the most played augmented reality game in history. With more than 44 million players at the peak of its popularity, the game has sparked interest on its effects on the young population’s health.

**Objective:**

This pilot study examined motivations to start playing Pokémon GO among a sample of US college students, and how motivations were associated with perceived outcomes of the playing experience and physical activity derived while playing.

**Methods:**

In November 2016, we asked a sample of 47 US college students (all Pokémon GO players) to complete online surveys and install an ecological momentary assessment (EMA) tool and step counter on their smartphones. The EMA tool prompted a set of questions on playing behavior and physical activity, 3 times per day (12:00 PM, 7:00 PM, and 10:00 PM), for 7 days. We used a factorial analysis to identify 3 distinctive groups of players based on their motivations to start playing Pokémon GO. We tested differences across motivation groups related to 5 unique outcomes using 1-way analysis of variance.

**Results:**

We extracted 3 interpretable factors from the clustering of motivations to start playing Pokémon GO: *Pokémon and video game fans* (n=26, 55% of the sample), *physical activity seekers* (n=8, 17%), and *curious & social* (n=13, 28%). The clusters differed significantly on the enjoyment of different aspects of the game, particularly battling, discovering new places, and meeting new people, as well as differences in agreement that playing improved mood and made them more social. Days when playing Pokémon GO were associated with higher number of steps reported at the end of the day, especially among *physical activity seekers*, but also for *Pokémon and video game fans*. All groups perceived traffic as a major threat to playing.

**Conclusions:**

Days during which Pokémon GO was played were positively associated with a set of beneficial health behaviors, including higher physical activity levels, more socialization, and better mood. Results, however, depended on personal motivations and expectations when joining the game. These results highlight the importance of taking motivation into account when attempting to extract conclusions from the Pokémon GO phenomenon to enhance future exergames’ designs or health interventions.

## Introduction

Worldwide, governments, health advocates, and public health researchers have been exploring programs and interventions to promote regular physical activity [1]. Despite this, physical activity levels have been declining worldwide. Adults and children in many countries around the world are not reaching recommended amounts of physical activity [2]. Early research suggests that playing Pokémon GO appears to increase daily physical activity levels, particularly among groups that have low levels of activity: teens, preteens, and younger adults [3].

Pokémon GO is a free-to-play, location-based augmented reality mobile game based on the popular video game series. The game was launched by Niantic, Inc in collaboration with Nintendo Co, Ltd in July 2016 in both Apple Inc’s iOS App Store and Google Inc’s Play Store. It involves capturing virtual characters, or Pokémon, that have been placed in prominent, real public locations, such as streets, parks, and other notable public buildings and spaces. The game allows players to create their virtual avatar and uses a mobile device’s global positioning system capability to display the player’s current geographic location on a map.

Pokémon GO is one among several recent augmented reality games that offer new ways to interact with the physical surroundings using a smartphone [4]. The physical activity component of the game, which requires players to walk and explore their surroundings using a detailed map of their neighborhood, led to several research opportunities on the potential benefits of Pokémon GO for youth and millennials. Among potential benefits, physical activity has been the most cited [4-8], as the active lifestyle promoted by Pokémon GO is seen as a potential intervention to tackle the obesity and inactivity epidemic [9,10]. What makes Pokémon GO particularly powerful and different from other kinds of exergames [11] is its attraction to people through game mechanics, making physical activity a secondary, or unintentional, behavior. The motivation and engagement of Pokémon GO players thus come not from turning physical activity into a game, but from a game with its own purpose that incorporates being active as part of the gaming experience.

Together with physical activity and motor skills, the game encourages players to explore their local communities and in the process may introduce them to new spaces [8]. This has led some to believe that Pokémon GO increases location awareness and public space use, and thus increases opportunities for social interaction within these spaces [12]. This capacity to increase social capital and establish new social networks through playing while being outside is also promising for mental health benefits [13,14], as social engagement linked to online gaming has been found to decrease perceived loneliness and depression [15]. In the particular case of Pokémon GO, the lack of an in-game chat app—a feature that other exergames include—makes players engage with one another via face-to-face communication and eliminates any possible alternatives via text messaging, which may be altering the socialization patterns usually seen in other online exergames. Finally, other potential outcomes may include better intergenerational relationships, as parents and their children engage together in outdoor activities [13], and increased knowledge of local patrimonial locations.

The rapid popularization of the game, which makes it the first augmented reality game with real global acceptance [16], has also raised some awareness of the risks players are creating or encountering [17,18]. These are mainly focused on the addictive nature of the game [19] and injury risk to players [20]. However, these are not risks associated only with Pokémon GO, as the risk of addiction has long been associated with some types of online video games [17], and injury risk is often a product of having distracted players in flawed public spaces that have long neglected pedestrians [12].

Overall, the game has many potential benefits that need proper research for validation and future interventions. To our knowledge, only a handful of studies have investigated the effects of playing Pokémon GO, establishing some links between playing and physical activity [4,5,21], and playing and risky behavior [12]. However, other aspects relevant to Pokémon GO’s popularity and public health remain completely unexplored. Among the most relevant are establishing what motivates people to begin and continue playing (or interacting with) the game, what their expectations and intentions are while playing, and whether the playing experience meets these expectations and intentions. These questions are particularly important if we want to use gaming to improve future health interventions. In that regard, and before Pokémon GO appeared, Tong et al [22] demonstrated how individuals respond differently to various gamification strategies. More recently, Chia-Chen and Liu [23] demonstrated that Pokémon GO players are far from being a unified group when it comes to playing motivations. Part of the huge popularity of the game relies on its attraction for people seeking different kinds of experiences. While first adopters might overwhelmingly be original Pokémon fans drawn to the game by their love of the franchise characters, once the popularity of the game reached a critical mass, many other people joined seeking either the social aspect or the exploration experience, or even out of curiosity for the game or the phenomenon itself. Distinguishing the drivers that lead different people to the game, and their staying power, is thus crucial to understand player behavior and preferences. Together with this, research in media psychology demonstrates how attitudes and experiences that motivate people to take part in a game can also modify the outcomes of such games [16].

This study aimed to fill the gap in knowing why players feel attracted to Pokémon GO, together with understanding the particular aspects of the game that best fulfill their motivations. We aimed to examine whether motivations correlated with perceived outcomes of playing and perceived risks encountered when playing Pokémon GO. Finally, we intended to use step counts derived from smartphone data to examine whether physical activity levels attained while playing Pokémon GO would fluctuate dependent on the motivations that led each specific player to the game. Findings on players’ behavior will inform future interventions through active games and exergames, enabling developers to design and tailor future games to target specific needs.

## Methods

### Recruitment

We recruited participants using the university recreation listserv of North Carolina State University, Raleigh, North Carolina, USA, with more than 8000 undergraduate student email addresses. The initial message stated the aim of the project and invited undergraduate students to participate in a study involving use of Pokémon GO and physical activity. We invited both players and nonplayers to participate. We offered entrance in a raffle of 8 gift cares worth US $50 as an incentive for those completing all required steps in the study, which included 2 surveys and the completion of 7 days of ecological momentary assessment (EMA). Recruitment occurred in early November 2016. After 1 week, we posted the same email message in an email from the student body president as part of a digest email including other topics. No additional recruitment efforts were included. All research protocols have been approved by North Carolina State University (Institutional Review Board no. 9242). We obtained written consent from all participants and stored the collected data on restricted computers in password-protected files.

### Study Design

Participants were asked to answer an initial online survey to collect demographics, weekly physical activity, and activity habits regarding smartphone, video game, and social networks use, together with their Pokémon GO playing habits. We assessed physical activity using the International Physical Activity Questionnaire (IPAQ) Short Form [24]. We then converted the metabolic equivalent tasks (METs) reported through the IPAQ questionnaire into kilocalories of energy expenditure through the formula 1 MET=1 kcal/kg/h [25]. We determined Pokémon GO playing habits through questions regarding playing frequency (number of days per week), time (played minutes per day), and time management (how they managed their playing time). Participants were also asked to download a step-counting app (PACER; Pacer Health Inc) to their smartphones together with an EMA app ([PACO]; Paco Developers). PACER [26] is one of many step counters that use smartphone-enabled accelerometers to estimate daily steps. We chose PACER due to its simplicity and low battery consumption. PACO [27] was developed as a basic EMA tool for research and has been previously used in studies [28]. Over a 7-day period, the PACO app would prompt a brief questionnaire 3 times per day (12:00 PM, 7:00 PM, and 10:00 PM). Questions asked about playing behavior during the preceding time period, including whether they had played, where they had played, with whom they had played, how they travelled to the playing site, and in which environment they had played. The final question on the PACO questionnaire involved reporting the actual number of steps recorded by the step counter.

At the end of the 7-day period, we asked participants who self-identified as Pokémon GO players to answer a final online survey with information regarding their motivations and perceived outcomes from playing Pokémon GO. Participants who completed a minimum of 80% of the potential EMA responses were entered in a raffle for 1 of 4 gift cards worth US $50.

### Statistical Analysis

To capture the multiple factors encouraging people to play Pokémon GO, we used a factor analysis to sort the underlying motivations to start playing Pokémon GO. Factor analysis reduces the number of input variables to a manageable number. Using a multiple response question list of 9 factors from which participants could choose to answer as many as they liked, we extracted 3 interpretable factors that exhibited the clustering of motivation for different types of players. We chose to retain the 3 factors with eigenvalue >1. Principal axis factoring and varimax rotation were used in deriving the results. We used means and standard deviations, together with 1-way analysis of variance (ANOVA), to analyze the differences between group scores in several different outcomes. Outcomes included in the analysis were enjoyment with different aspects of Pokémon GO (measured on a Likert scale ranging from 1 to 4); spatial awareness while playing (1-4 Likert scale); and threats encountered while playing (multiple responses from a list). Finally, we also considered media effects by asking how playing Pokémon GO had made players more physically active, improved their mood, and increased social interactions (1-4 Likert scale). We used 1-way ANOVA and chi-square tests to test differences in means and distributions. We used post hoc Tukey tests on those variables with statistically significant difference in group means to confirm where the differences occurred between groups. Finally, between- and within-participants 1-way ANOVA were used to analyze differences between groups on the total amount of physical activity recorded at the end of the playing or nonplaying days (time=10:00 PM).

## Results

### Study Population

A total of 123 students replied to our emails expressing interest to participate. Because we did not know the actual number of email recipients actually reading the recruitment text, we were unable to calculate an accurate response rate. The overall study recruited both players and nonplayers. Among the final sample of 123 respondents, 49 had to be discarded because either they didn’t answer the first online survey or they provided insufficient EMA data. From the 74 remaining participants, 27 identified as nonplayers during the initial online survey and 47 identified as players.

The study population (n=47) included 24 males with an average age of 19.8 years, and 23 females with an average age of 19.1 years. The most frequent ethnicity was white (n=33) followed by Asian (n=10). Most participants where between their freshman (n=21) and sophomore year (n=14). All of the participants had experience playing Pokémon GO prior to the start of the study. They had been playing Pokémon GO for a median of 122 days (SD 18.1) before the start of the study.

### Factor Analysis

Together, the list of factors obtained through the factor analysis accounted for 60.5% of the variance among the 9 motivation variables listed (Table 1). The first factor scored highly on “I’m a Pokémon fan” and “I like video games,” which we collectively grouped into *Pokémon and video game fans*. They likely represent the first wave of players, attracted by the contents of the game itself. The second factor scored highly on “to walk more” and “for exercise.” We grouped these as *physical activity seekers* and hypothesized they were attracted to the game by the numerous reports that claimed playing was beneficial for physical activity. Finally, the third factor scored highly on a variety of answers, unrelated to the physical activity or the contents of the game. In particular, the scores were higher for “To learn about my city” and “To meet new people,” with curiosity about the technology and the phenomenon itself also scoring highly. We referred to these as the *curious & social*.

**Table 1:** Analysis loadings and summary for factors encouraging people to play Pokémon GO^a^.

Why did you start playing Pokémon GO?		Factor					
		1. Pokémon and video game fans (n=26)		2. Physical activity seekers (n=8)		3. Curious & social (n=13)	
For exercise				.716			
To walk more				.872			
I was curious about the technology						.575	
I was curious about people playing						.589	
To learn about my city						.755	
I’m a Pokémon fan		.822					
I like video games		.599					
To meet new people						.754	
I was encouraged by others						.680	
**Summary statistics**							
	Eigenvalue		8.6%		15.5%		35.3%
	Initial eigenvalue		1.12		1.82		3.54
	Percentage of variance^b^		12.4%		20.3%		39.2%

^a^Only loadings >0.40 are shown. Keiser-Meyer-Olkin=.682; Bartlett <0.001.

^b^Cumulative percentage of variance captured by factors = 60.5%.

There were no significant differences in the demographic composition of each cluster, as Table 2 shows. *Pokémon and video game fans* tended to be male, with the highest proportion of Asian American students. Although differences were not significant, *Pokémon and video game fans* tended to be less active (having the lowest energy expenditure in kcal/week reported among the 3 groups, at 2850 kcal). This group reported playing between 4 and 5 days per week, accumulating 120 minutes of play per week. The *physical activity seekers* showed an equal balance between sexes, were predominantly white, and were the youngest of the 3 groups, being either freshman or sophomore in class standing (generally 18-20 years of age). They were also the most active group overall, but reported playing only 3 days per week for a total of 59 minutes per week. Finally, the *curious & social* group were predominantly white females and 20 years of age on average. They reported almost 6 days of play per week and logged a total 120 minutes of play per week.

Of all analyzed variables, only the accumulated experience playing Pokémon GO at the start of the study was significantly different between groups. A post hoc test (Tukey) showed that the *physical activity seekers* differed significantly (*P*<.001) from the 2 other groups in the accumulated gaming experience before joining the study. While *Pokémon and video game fans* and *curious & social* players started playing Pokémon GO shortly after the launch of the game (accumulating a median of 122 and 123 days of experience, respectively, before starting the study), *physical activity seekers* were drawn to the game much later and had only 100 days of experience at the start of the study.

### Gaming Experience and Perceptions

The different underlying motivations to play Pokémon GO are reflected in both the gaming experience and the perceived behavioral outcomes of the game. Table 3 displays players’ perceptions regarding the factors in terms of what aspects they enjoyed and their perceived threats experienced while playing. Players could select as many enjoyment factors as they wanted and, on average, catching Pokémon and playing along with friends were the highest-rated aspects of the game. These scores, however, varied across motivation clusters. The clusters differed significantly on the rating of battling (χ^2^_(2,n=47)_=15.4, *P*<.001), discovering new places (χ^2^_(2,n=47)_=11.5, *P*=.003), and meeting new people (χ^2^_(2,n=47)_=20.9, *P*<.001). In all 3 cases, post hoc tests revealed that the *curious & social* group had a significantly higher rating. While *Pokémon and video game fans* and *physical activity seekers* rated these 3 particular aspects very low, these were highly valued items for the enjoyment of the *curious & social* group.

The 3 groups did not differ significantly on encountering threats. Nearly half of the players (49%) reported encountering some kind of threat while they were playing, with the presence of traffic (n=16, 34%) and poor walking conditions (n=14, 30%) being the most frequent.

**Table 2:** Demographic characteristics and play averages per group of factors.

Characteristics		All (n=47)		Factor									Chi-square test		
				1. Pokémon and video game fans (n=26)		2. Physical activity seekers (n=8)			3. Curious & social (n=13)						
												*F*			*P* value
**Sex, n (%)**											1.243			.54	
	Female	23 (49%)		11 (42%)		4 (50%)		8 (62%)							
	Male	24 (51%)		16 (57%)		4 (50%)		5 (39%)							
**Ethnicity, n (%)**											1.244			.54	
	White	33 (70%)		18 (69%)		5 (63%)		10 (77%)							
	Asian	10 (21%)		8 (31%)		0 (0%)		2 (15%)							
	Other	4 (9%)		0 (0%)		3 (38%)		1 (8%)							
**Academic standing, n (%)**											2.985			.56	
	Freshman	21 (45%)		13 (50%)		4 (50%)		5 (39%)							
	Sophomore	14 (30%)		7 (27%)		4 (50%)		4 (31%)							
	Junior or Senior	12 (26%)		6 (23%)		0 (0%)		4 (31%)							
**Analysis of variance**															
	Age (years), mean (SD)	19.5 (2.1)		19.3 (1.6)		19.0 (1.1)		20.1 (3.3)			0.77			.47	
	Energy expenditure (kcal/week)^a^, mean (SD)	3283.5 (1968.9)		2850.3 (1753.2)		4120.1 (1947.1)		3830.5 (2291.8)			1.78			.18	
	Play/week (minutes), median (IQR^b^)	154 (250)		120 (520)		59 (396)		120 (530)			1.03			0.37	
	Play/week (days), median (IQR)	5 (3)		5 (4.5)		3 (2)		6 (4)			0.22			.80	
	Playing experience (days)^c^, median (IQR)	122 (11)		122 (44)		100 (59.3)		123 (11)			11.674			<.001	

^a^Energy expenditure estimated by transforming metabolic equivalent tasks (METs) into kilocalories, where 1 MET=1 kcal/kg/h [25].

^b^IQR: interquartile range.

^c^Self-reported number of days playing Pokémon GO at the start of the study.

**Table 3:** Enjoyment elements and threats encountered per group of factors.

Questions		All (n=47)		1. Pokémon and video game fans (n=26)		2. Physical activity seekers (n=8)			3. Curious & social (n=13)			Chi-square test		
		n (%)		n (%)			n (%)			n (%)			χ^2^	*P* value
**What do you enjoy the most?**^a^														
	Catching Pokémon	42 (89%)		24 (92%)			7 (88%)			11 (85%)			0.9	.64
	Battling	22 (47%)		9 (35%)			1 (13%)			12 (92%)			15	<.001
	Playing with friends	33 (70%)		18 (69%)			4 (50%)			11 (85%)			2.5	.28
	Being outdoors	26 (55%)		12 (46%)			4 (50%)	5		10 (77%)			0.0	.98
	Discovering new places	25 (53%)		9 (35%)			4 (50%)			12 (92%)			11.5	.003
	Meeting new people	9 (19%)		1 (4%)			0 (0%)			8 (62%)			20.9	<.001
**Have you encountered any threats while playing?**^a^														
	Traffic	16 (34%)		8 (31%)			5 (63%)			3 (23%)			3.59	.17
	Poor walking conditions	14 (30%)		7 (27%)			4 (50%)			3 (23%)			4.1	.11
	None	24 (51%)		15 (58%)			1 (13%)			8 (62%)			3.7	.15

^a^Dichotomized results of multiple-choice answers.

Table 4 displays players’ perceived outcomes and changes in players’ spatial awareness. Respondents were asked to respond on 4-point Likert-type scales ranging from “not at all” to “to a great extent.” Players reported a very mild adherence to the idea that playing Pokémon GO had made them more physically active (mean score 2.89, SD 0.78). Differences were found in the statement regarding whether playing had improved their mood. On average, players did agree that playing had “somewhat” improved their mood. However, differences across groups were significant (*F*_2,45_=3.623, *P*=.04), with the *physical activity seekers* agreeing least with the statement (mean 2.5 out of 4, SD 0.84). Post hoc Tukey tests revealed the *curious & social* to score significantly higher (*P*=.008) than the *physical activity seekers* on improved mood. Finally, players on average stated that the game had done “very little” (mean 2.23 out of 4, SD 0.91) to improve their social interactions, with the highest rating among the *curious & social*, who significantly differed from the other 2 groups (*F*_2,45_=6.285, *P*=.004) and stated that the game had made them “somewhat” more social.

On average, players reported the game had made them a little more aware of their neighborhood, (mean 2.191, SD 1.08) and their surrounding facilities (mean 2.149, SD 1.02). Players also reported that playing had made them somewhat more aware of the public spaces around them (mean 2.776, SD 1.07). Significant differences were found in the reporting of neighborhood and facility awareness and, in all cases, *Pokémon and video game fans* had the least awareness, while the *curious & social* had the highest awareness.

**Table 4:** Outcome perceptions and spatial awareness per group of factors.

Questions		All (n=47)			1. Pokémon and video game fans (n=26)			2. Physical activity seekers (n=8)			3. Curious & social (n=13)			Analysis of variance	
		Mean (SD)		Mean (SD)			Mean (SD)			Mean (SD)			*F*		*P* value
**Has playing Pokémon GO…**^a^															
	Helped you be more physically active	2.89 (0.67)		2.86 (0.76)			2.67 (0.52)			3.08 (0.49)			0.875		.42
	Improved your mood	3.04 (0.589)		3.07 (0.54)			2.5 (.84)			3.23 (0.44)			3.623		.04
	Improved your social interactions	2.23 (0.91)		1.96 (0.88)			2.0 (0.89)			2.92 (0.64)			6.285		.004
**Has playing Pokémon GO made you aware of new…**^a^															
	Neighborhood	2.19 (1.08)		1.89 (0.96)			2.33 (1.21)			2.77 (1.09)			3.304		.046
	Public space	2.77 (1.07)		2.54 (1.2)			2.83 (1.17)			3.23 (0.44)			1.976		.15
	Facilities	2.145 (1.02)		1.86 (1.11)			2.17 (0.75)			2.77 0.60)			4.006		.03

^a^Answers were scored on a 4-point Likert scale: 1=not at all; 2=very little; 3=somewhat; 4=to a great extent.

**Figure 1:**
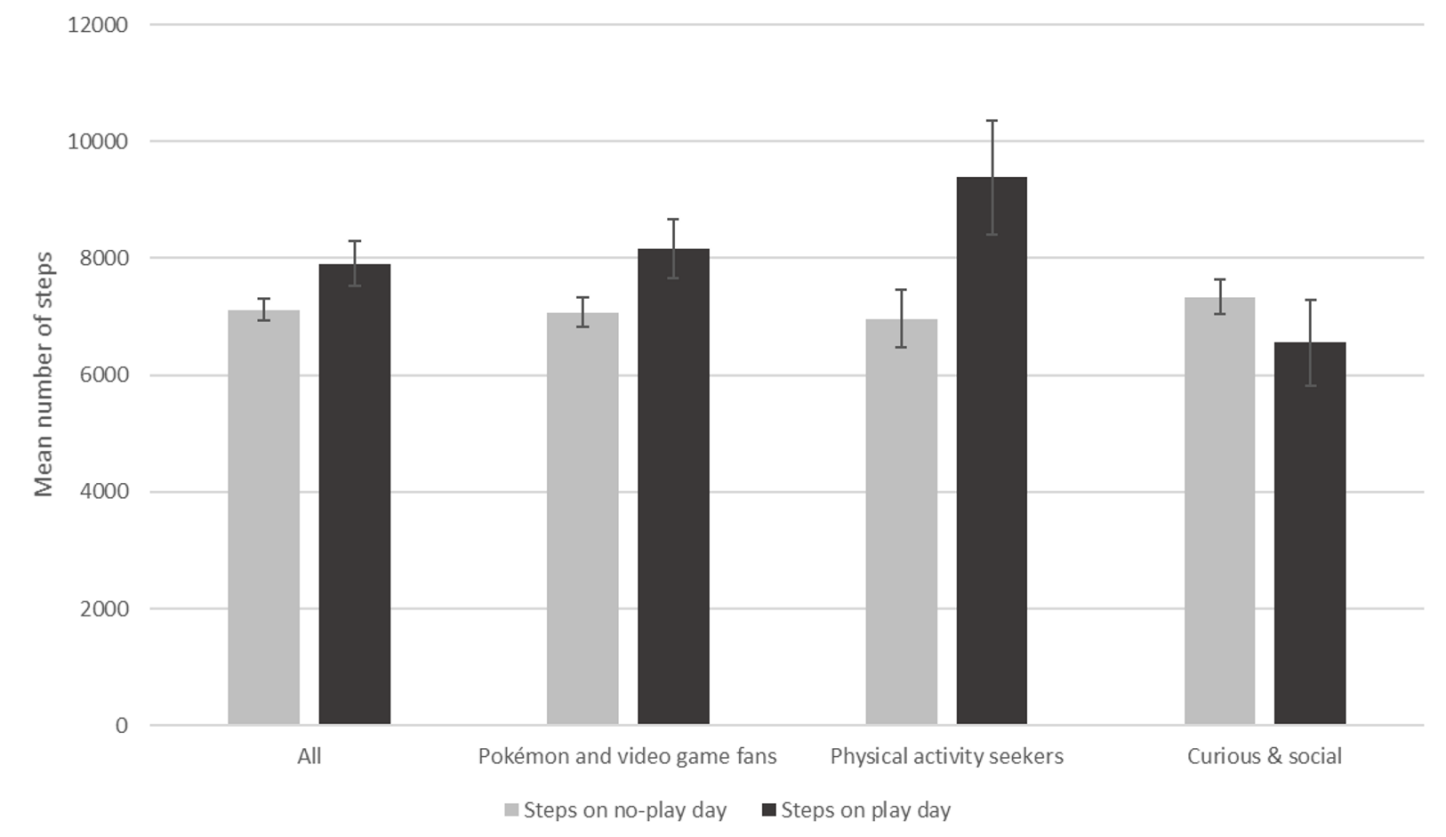
Daily steps measured at 10:00 PM on playing and nonplaying weekdays. Error bars indicate SD.

### Objectively Measured Physical Activity

Among the group of self-identified Pokémon GO players, a Pokémon GO playing day, versus a nonplaying day, was associated with higher number of steps reported (*F*_1,310_=8.903, *P*=.048; Figure 1). Among the different motivation groups, the relationship is clearly stronger among *physical activity seekers* (*F*_1,39_=0.887, *P*=.02), as they were reporting 34.76% more steps on playing days (n=9382) than on days when they had not played (n=6962). For *Pokémon and video game fans*, playing was also associated with a higher level of physical activity (*F*_1,195_=2.842, *P=*.04). Finally, the *curious & social* group reported fewer steps on playing days than in nonplaying days, although that difference was not significant.

## Discussion

### Principal Findings

This study found that Pokémon GO players were motivated to play for a variety of reasons, and these motivations determined their perceived and objective behavioral outcomes of playing. Understanding the drivers of playing behavior, along with factors associated with the satisfaction of playing Pokémon GO, is a central part of using the whole phenomenon of the Pokémon GO craze of 2016 to inform better future health interventions through gaming. Together with understanding the outcomes of playing Pokémon GO, research on playing behavior might contribute to designing more effective interventions to tackle sedentary behaviors, increase physical activity, and increase outdoor socialization among younger adults.

Despite having a limited sample size and focusing on a specific population—that is, undergraduate college students—our analysis provides objectively measured physical activity as an outcome of playing, together with the use of an EMA tool to enhance the monitoring of motivations and perceptions of players. These results constitute a step forward toward empirically measuring the causes that have driven numerous players to Pokémon GO, together with objective measures of their playing experience.

Our results distinguished 3 unique groups from a sample of US college students drawn to Pokémon GO: explicitly seeking physical activity; being Pokémon fans; and being curious about the phenomenon and wanting to explore opportunities to socialize. Belonging to each group was associated with a different set of enjoyment factors and variations of perceived outcomes derived from playing. Due to Pokémon GO’s novelty and trendiness, little research has been done on either the determinants or the outcomes of playing Pokémon GO. Until now, to our knowledge, only the study by Rasche et al [29] has assessed players’ motivations. Consistent with their findings, this study also found that being a fan of Pokémon was the most frequent motivation to start playing. Undergraduate students are among the intended target of the game, being already familiar with the Pokémon world. According to Bonus et al [16], these players might have been attracted to the game by the power of a retro brand like the Pokémon franchise, stimulating their interest in the new product by triggering nostalgic and positive emotional attachments. Our data, however, also found a specific group of players drawn to the game by the social aspect and another that mainly sought physical activity. Interestingly, this latter group was also the group that joined Pokémon GO later (with only 100 days of playing experience prior to joining the study). This might indicate that it took people reading or hearing about the physical activity benefits of the game reported in the press to become involved in Pokémon GO. This is also consistent with the idea that peer and social pressure in the form of encouragement from family and friends, together with praise from the press, might influence an individual’s adoption of the game [16].

Our data clearly suggest that motivations and exposure effectively determined a player’s response and outcomes. For instance, those who started playing because they were Pokémon fans enjoyed Pokémon-related characteristics of the game, but also playing with friends; those seeking physical activity enjoyed catching Pokémon; and those *curious & social* enjoyed the more social aspect of playing Pokémon GO (battling), together with playing with friends and making new discoveries. Playing alongside friends was, in fact, the highest-rated aspect, which also emphasizes the need for considering the social dimension in future games or interventions.

Significantly, *physical activity seekers* were the ones in least agreement with the idea that playing Pokémon GO could improve their physical activity levels. Members of the other groups, however, were more in agreement with Pokémon GO having increased their physical activity. This suggests that Pokémon GO may have a positive secondary outcome on increasing physical activity, but that people seeking exclusively physical activity may end up disappointed due to the limited increase in steps. The most agreed-upon assessment among all groups was that playing Pokémon GO improved their mood. This could have been a consequence of being physically active [30], being outdoors, or even staying focused on a single task while avoiding social media [31]. Also significant is that those who joined looking for increased social interactions (*curious & social*) were the ones most in agreement with the idea that Pokémon GO had improved their social interactions. This may validate the idea that playing Pokémon GO has some positive socialization effects, an idea that has been repeatedly posed by experts [7] but that has not yet, to our knowledge, been tested empirically. Our results also support the idea that Pokémon GO may increase spatial exploration and location awareness. While Pokémon GO fans paid little attention to their location, awareness of surroundings was highly appreciated by those who were playing because they were curious, or for social or physical activity, with the discovery of new public places being the most cited feature. Not paying attention to the surroundings could also be a risk factor, as players can become so focused on the playing experience that they put themselves in danger [12]. However, our study did not ask specifically about risky playing behaviors.

The study’s group of players reported higher activity levels on days when they had actually played Pokémon GO. Playing also had different physical activity implications depending on the motivations of each player. Based on steps reported through smartphone accelerometers, our results suggest an 11.05% difference in the number of daily steps between active playing days (n=7909) and nonplaying days (n=7122). This effect of Pokémon GO on physical activity, however, was not homogeneous across all groups. Pokémon fans registered a 15.37% increase in steps on playing days (from 7077 steps on nonplaying days to 8165 steps on playing days), while for the *physical activity seekers* this increased by 34.76% (from 6962 steps on nonplaying days to 9382 steps on playing days). The *curious & social*, by contrast, reported a 10.71% reduction of steps during playing days (from 7338 steps on nonplaying days to 6552 on playing days). The overall finding is consistent with the only other available study using objective counts of physical activity [21], which found a 22.4% step increase among Pokémon GO users considering only the first week after installing the game. Our motivation-specific results reinforce the idea that physical gains derived from Pokémon GO are not universal, as different player groups, with different motivations and expectations, may be getting different levels of physical during and because of their playing experience. Other analyses using self-reported measures of physical activity, with less accuracy but larger sample sizes, also reported slight increases in physical activity [3] and that Pokémon GO was particularly popular among people with sedentary behaviors [5].

Previous research [12] reported that increased socialization and a higher number of visits to green areas and public parks were among the most often reported benefits of the Pokémon GO phenomenon. Our results may contradict this and other exergame examples [32,33], as we did not find an association between those players who were more motivated by social connections and increased physical activity.

### Limitations

Despite the use of EMA and objective physical activity through the smartphone accelerometer, this study has limitations. First and foremost, our small sample size limits the conclusions that can be drawn from this study. In the future, extending the study to a larger population, other age groups, and additional locations should validate and confirm the conclusions we reached. While the use of EMA tools provided higher accuracy on the collected data, it also made it necessary to keep surveys, which were administered 3 times a day, reasonably short in order to avoid low response rates. Not enough data were available from those who did not complete the EMA section of the study in order to compare their results with those who successfully completed it. With the short EMA surveys, we still removed 7 participants from the analyses because they did not complete 80% of the 21 surveys.

### Conclusions

Bielik et al [34] described 10 design requirements that make exergames successful at promoting physical activity. Of those, Pokémon GO meets 6. (1) *Personal awareness of activity levels* and (2) providing *feedback on the accumulated activity* are met in that Pokémon GO players always know how many steps they have taken and how many times they have played. Pokémon GO also (3) *supports social influences*, both by allowing the sharing of in-game achievements with others and by promoting spontaneous social interactions. It also (4) provides a *variety of motivational tools* that, as shown by our results, range from playing with the goal of catching Pokémon or playing with the goal of engaging in between-player battles, to playing for exercise or playing to socialize. This wide range of motivational tools creates (5) *short- and long-term incentives* for Pokémon GO players to leave home and engage in physical activity. Pokémon GO does this while (6) *understanding the practical constraints of each player’s* lifestyle and allowing important freedom regarding schedules and playing bouts. Bielik and colleague’s requirements that are not completely met by Pokémon GO are *giving the user proper credit for [physical] activity*; *ensuring fair play*; providing the *possibility of integrations with other existing solutions*; and, finally, meeting the issue of *data privacy* and how developers are protecting players’ data.

Previous research had already demonstrated the positive effect that exergames can have on physical activity among young people [35,36], at the same time that others have demonstrated the impact of social online video games on the development of social capital in the form of new and stronger friendships [37]. The strong links in the literature between social interaction and exercise [38] make it plausible that the effects of Pokémon GO go beyond the mere increased physical activity and enhanced socialization toward a broader concept of mental health and well-being [16]. In particular, what makes Pokémon GO different and more effective than similar kinds of exergames are its social component and the special use of augmented reality that transforms the actual physical surrounding environment into a digital playing field.

Although the direct observed benefits of playing were small, the success of Pokémon GO can encourage future health interventions using active games and exergames. We have demonstrated the potential of this kind of game to increase physical activity levels among US college students. At the same time, we have also demonstrated how the effects were not homogeneous among all players, as motivations and personal attitudes toward the game can change the perceived and objective outcomes. The fact that Pokémon GO is gathering people seeking different experiences is a positive indicator and a note to future interventions that should plan for a broader range of players.

Understanding how to design games to bring together public health interventions will be important in a future with an ever greater presence of mobile games and virtual reality experiences. This study provides emerging evidence to validate some of the suggested positive outcomes of playing Pokémon GO and contributes to understanding the complexity behind gaming behavior and gaming experience. Further insights into how to engage players in even more active playing behaviors, along with new understandings on how to maintain long-term user engagement, will be necessary for the future. Finally, qualitative studies and mixed-methods approaches will be necessary in the future to deepen the conclusions advanced here.

## Abbreviations

ANOVA: analysis of variance
EMA: ecological momentary assessment
IPAQ: International Physical Activity Questionnaire
MET: metabolic equivalent task
PACO: Personal Analytics Companion
